# Supplementary material for: Altered heparan sulfate metabolism during development triggers dopamine-dependent autistic-behaviours in models of lysosomal storage disorders
Source: Nat Commun. 2021 Jun 9;12:3495. doi: 10.1038/s41467-021-23903-5 (PMC8190083; doi:10.1038/s41467-021-23903-5)
Supplement: Supplementary file 1 — Supplementary information. [file 41467_2021_23903_MOESM1_ESM.pdf]

SUPPLEMENTARY INFORMATION

Altered heparan sulfate metabolism during development triggers dopamine-dependent autistic-behaviours in models of lysosomal storage disorders

De Risi et al.,

SUPPLEMENTARY TABLES

	Object 1	Object 2
**WT**	191.3 ± 24.5	150.3 ± 14.4
**MPS-IIIA**	176.6 ± 24.1	163.9 ± 22.3

Supplementary Table 1. No differences were observed in the total time of exploration during the habituation phase of the social novelty preference task. Data presented as mean ± S.E.M.

Main Figure	Statistical test	Sample size
**1a'**	One-way ANOVA (Factor: genotype, 2 levels: WT, MPS-IIIA)<br>2 months: $F_{1,15}$=6.877; p=0.01; 8 months: $F_{1,16}$=16.199; p=0.001	2-months<br>WT=9, MPS-IIIA=8;<br>8-months<br>WT=8, MPS-IIIA=10
**1a''**	One-way ANOVA (Factor: genotype, 2 levels: WT, MPS-IIIA)<br>2 months: $F_{1,15}$=5.137; p=0.03; 8 months: $F_{1,16}$=15.371; p=0.0012	
**1b**	One-way ANOVA (Factor: genotype, 2 levels: WT, MPS-IIIA)<br>2 months: $F_{1,15}$=6.628; p=0.02	
**1b'**	One-way ANOVA (Factor: genotype, 2 levels: WT, MPS-IIIA)<br>2 months: $F_{1,15}$=8.923; p=0.0092	
**1c**	χ2 test<br>2 months: 0.009	
**1c'**	One-way ANOVA (Factor: genotype, 2 levels: WT, MPS-IIIA)<br>2 months: $F_{1,15}$=8.509; p=0.01	
**1d**	One-way ANOVA (Factor: genotype, 2 levels: WT, MPS-IIIA)<br>8 months: $F_{1,16}$=24.695; p=0.0001	
**1 e**	Repeated measures ANOVA (2 levels: training and test; Factor: genotype, 2 levels: WT and MPS-IIIA)<br>8 months: Genotype $F_{1,16}$=2.729; p=0.11; % freezing $F_{1,16}$=34.214; p=0.0001; Genotype x % freezing $F_{1,16}$=11.401; p=0.0038	
**2b**	One-way ANOVA (Factor: genotype, 2 levels: WT, MPS-IIIA)<br>$F_{1,11}$=18.949; p=0.001	WT=7, MPS-IIIA=6
**2c**	One-way ANOVA (Factor: genotype, 2 levels: WT, MPS-IIIA)<br>$F_{1,7}$=8.83; p=0.02	WT=4, MPS-IIIA=5
**2d- d'**	One-way ANOVA (Factor: genotype, 2 levels: WT, MPS-IIIA)<br>$F_{1,11}$=6.33; p=0.02	WT=6-7, MPS-IIIA=5-6
**2e-e'**	Repeated measures ANOVA (3 levels: nACC, DMS, DLS; Factor: genotype, 2 levels: WT and MPS-IIIA)<br>D1R: Genotype $F_{1,12}$=51.686; p=0.0004; Subregion $F_{2,12=}$ 9.491; p=0.0034; Genotype x Subregion $F_{2,12=}$ 9.491; p=0.0034<br>D2R/SYN: Genotype $F_{1,12}$=426.4; p<0.0001; Subregion $F_{2,12=}$ 0.009; p=0.99; Genotype x Subregion $F_{2,12=}$ 0.009; p=0.99<br>D2R/PSD-95: Genotype $F_{1,12}$=7.405; p=0.03; Subregion $F_{2,12=}$ 0.711; p=0.51; Genotype x Subregion $F_{2,12=}$ 0.711; p=0.51	WT=4, MPS-IIIA=4
**2f-f'**	Two-way ANOVA (Factor: genotype, 2 levels: WT, MPS-IIIA; Factor: treatment, 3 levels: vehicle, SCH and halo)<br>P-DARPP-32: Genotype $F_{1,27}$=6.79; p=0.01; Treatment $F_{2,27}$=28.7; p<0.0001; Genotype x treatment $F_{2,27}$=3.13; p=0.05	WT veh=8, MPS-IIIA veh=7; WT SCH=4; MPS-IIIA SCH=6; WT halo=4; MPS-IIIA halo=4
**3a-a''**	Two-way ANOVA (Factor: genotype, 2 levels: WT, MPS-IIIA; Factor: treatment, 2 levels: vehicle, α-MPT) or χ2 test<br>Distance: Genotype $F_{1,21}$=6.930; p=0.01; Treatment $F_{1,21}$=22.280; p<0.0001; Genotype x treatment $F_{1,21}$=4.61; p=0.04)<br>Grooming: Genotype $F_{1,21}$=10.38; p=0.004; Treatment $F_{1,21}$=11.28; p=0.003; Genotype x treatment $F_{1,21}$=4.59; p=0.04<br>Rearing: Genotype $F_{1,21}$=1.02; p=0.32; Treatment $F_{1,21}$=7.35; p=0.01; Genotype x treatment $F_{1,21}$=12.45; p=0.0019<br>Social tube: χ2=0.003	WT veh= 6, MPS-IIIA veh=7, WT α-MPT=6, MPS-IIIA α-MPT= 6
**3b-b''**	Two-way ANOVA (Factor: genotype, 2 levels: WT, MPS-IIIA; Factor: treatment, 3 levels: vehicle, SCH 0.03, SCH 0.06) or χ2 test<br>Distance: Genotype $F_{1,52}$=2.15; p=0.14; Treatment $F_{2,52}$= 5.33; p=0.007; Genotype x Treatment $F_{2,52}$=4.27; p=0.01	WT veh= 14; MPS-IIIA veh=15; WT SCH 0.03=6; MPS-IIIA SCH 0.03=8;

	Rearing: Genotype $F_{1,52}$=3.74; p=0.05; Treatment $F_{2,52}$= 5.18; p=0.008; Genotype x Treatment $F_{2,52}$=4.31; p=0.01<br>Grooming: Genotype $F_{1,52}$=2.56; p=0.11; Treatment $F_{2,52}$= 3.13; p=0.05; Genotype x Treatment $F_{2,52}$=4.28; p=0.01<br>Sociability: Genotype $F_{1,52}$=3.27; p=0.07; Treatment $F_{2,52}$=2.22; p=0.11; Genotype x Treatment $F_{2,52}$=8.25; p=0.0007<br>Social tube: χ2<0.05	WT SCH 0.06=8; MPS-IIIA SCH 0.06=7
**3c-c''**	Two-way ANOVA (Factor: genotype, 2 levels: WT, MPS-IIIA; Factor: treatment, 3 levels: vehicle, halo 0.05, halo 0.1) or χ2 test<br>Distance: Genotype $F_{1,39}$=15.655; p=0.0003; Treatment $F_{2,39}$= 8.137; p=0.0011; Genotype x treatment $F_{2,39}$=3.34; p=0.04<br>Rearing: Genotype $F_{1,39}$=21.89; p<0.0001; Treatment $F_{2,39}$=0.050; p=0.95; Genotype x treatment $F_{2,39}$=2.63; p=0.08<br>Grooming: Genotype $F_{1,39}$=15.025; p=0.0004; Treatment $F_{2,39}$=0.027; p=0.97; Genotype x treatment $F_{2,39}$=1.19; p=0.31<br>Sociability: Genotype $F_{1,39}$=24.388; p<0.0001; Treatment $F_{2,39}$=0.166; p=0.84; Genotype x treatment $F_{2,39}$=1.75; p=0.18	WT veh= 8; MPS-IIIA veh=7; WT halo 0.05=8; MPS-IIIA halo 0.05=7; WT halo 0.1=8; MPS-IIIA halo 0.1=7
**4a**	Repeated measures ANOVA (2 levels: SN and VTA; Factor: genotype, 2 levels: WT and MPS-IIIA)<br>Genotype $F_{1,7}$=9.106; p=0.01; Subregion $F_{1,7}$=0.251; p=0.6; Genotype x Subregion $F_{1,7}$=0.251; p=0.6	WT=4; MPS-IIIA=5
**4a'**	One-way ANOVA (Factor: genotype, 2 levels: WT, MPS-IIIA)<br>$F_{1,7}$=15.959; p=0.005	
**4b**	One-way ANOVA (Factor: genotype, 2 levels: WT, MPS-IIIA)<br>E13.5: $F_{1,6}$=8.948; p=0.024<br>P0: $F_{1,5}$=9.436=0.02<br>Repeated measures ANOVA (2 levels: SN and VTA; Factor: genotype, 2 levels: WT and MPS-IIIA)<br>Genotype $F_{1,5}$=14.004; p=0.01; Subregion $F_{1,5}$=0.179; p=0.60; Genotype x Subregion $F_{1,5}$=0.179; p=0.60	WT=3-4; MPS-IIIA=3-4
**4d-e**	One-way ANOVA (Factor: genotype, 2 levels: WT, MPS-IIIA)<br>BrdU: $F_{1,6}$=6.050; p=0.04<br>LMX1A: $F_{1,4}$ =38.491; p=0.0034	WT=3-4; MPS-IIIA=3-5
**5b-c**	Two-way ANOVA (Factor: genotype, 2 levels: WT, MPS-IIIA; Factor: DIV; 3 levels: DIV4, DIV7, DIV14)<br>TH: Genotype $F_{1,23}$=5.045; p=0.03; DIV $F_{2,23}$=2.853; p=0.07; Genotype x DIV $F_{2,23}$= 25.517; p<0.0001<br>MAP2: Genotype $F_{1,14}$=28.554 p=0.0001; DIV $F_{2,14}$=13.79; p=0.0005; Genotype x DIV $F_{2,14}$= 24.165; p<0.0001	WT n=3-4; MPS-IIIA n=4-8
**6a'-b-c-d**	One-way ANOVA (Factor: genotype, 2 levels: WT, MPS-IIIA)<br>TH+ cells DIV4: $F_{1,5}$=225.558; p<0.0001<br>DA release DIV4: $F_{1,5}$=34.239; p=0.021<br>MTS DIV4: $F_{1,6}$=57.569; p=0.0003<br>BrdU DIV4: $F_{1,4}$=27.380; p=0.0064	WT DIV1 n=3-4; MPS-IIIA DIV1 n= 3-4; WT DIV4 n=3-4; MPS-IIIA DIV4 n=3-4
**7a'-a''-a'''**	One-way ANOVA (Factor: transfection, 3 levels: WT, MPS-IIIA, MPS-IIIA + *SGSH*-Flag)<br>TH+ cells: $F_{2,7}$=60.202; p<0.0001<br>MTS: $F_{2,9}$=165.268; p<0.0001<br>BrdU: $F_{2,9}$=333.268; p<0.0001	WT n= 3-4; MPS-IIIA n=3-4; MPS-IIIA + SGSH-Flag n=4
**7b'-b''-b'''**	Two-way ANOVA (Factor: genotype, 2 levels: WT, MPS-IIIA; Factor: Treatment; 3 levels: veh, HS 25, HS40)	WT n=3-4; WT HS25 n=3-4; WT HS40

	TH+ cells: Genotype $F_{1,13}$=581.143; p<0.0001; Treatment $F_{2,13}$=10.926; p=0.0016; Genotype x treatment $F_{2,13}$=10.330; p=0.0021<br>MTS: Genotype $F_{1,17}$=236.045; p<0.0001; Treatment $F_{2,17}$=17.911; p<0.0001; Genotype x treatment $F_{2,17}$=6.471; p=0.0081<br>BrdU: Genotype $F_{1,12}$=247.317; p<0.0001; Treatment $F_{2,12}$=20.655; p=0.0001; Genotype x treatment $F_{2,12}$=21.074; p=0.0001	n=3; MPS-IIIA n=3-4; MPS-IIIA HS25 n=3-4; MPS-IIIA HS40 n=3-4
**8a-a'; 8b-b'; 8c'; 8d; 8f-g'**	One-way ANOVA (Factor: genotype, 2 levels: WT, MPS-II)<br>Grooming: $F_{1,15}$=5.510; p=0.031<br>Rearing: $F_{1,15}$=10.874; p=0.0049<br>Sociability index: $F_{1,15}$=7.689; p=0.01	WT= 8; MPS-II=9
**8c**	$\chi 2$ test	
**8e**	Repeated measures ANOVA (2 levels: training and test, Factor: genotype, 2 levels: WT and MPS-II)<br>Genotype $F_{1,15}$=0.002; p=0.9; % freezing $F_{1,15}$=46.37; p<0.0001; Genotype x % freezing $F_{1,15}$=0.75; p=0.39	
**8f-g'**	One-way ANOVA (Factor: genotype, 2 levels: WT, MPS-II)<br>TH striatum: $F_{1,5}$=7.959; p=0.02<br>Repeated measures ANOVA (2 levels: SN and VTA; Factor: genotype, 2 levels: WT and MPS-II)<br>TH+ cells: Genotype $F_{1,5}$=22.958; p=0.0049; subregion $F_{1,5}$= 1.425 ; p=0.28; Genotype x subregion $F_{1,5}$= 1.425 ; p=0.28	WT=4-5, MPS-II=3-5

Supplementary Table 2. Detailed information for the main figures.

Supp. Figure	Statistical test	Sample size
**1a**	Repeated measures ANOVA (2 levels: familiar and new; Factor: genotype, 2 levels: WT and MPS-IIIA)<br>Genotype $F_{1,11}$=0.83; p=0.38; Time $F_{1,11}$=8.2; p=0.01; Genotype x Time $F_{1,11}$=0.40; p=0.53	2-months<br>WT=7, MPS-IIIA=6
**2a-a'; 2b-b'; 2c'; 2d**	One-way ANOVA (Factor: genotype, 2 levels: WT, MPS-IIIA)<br>Rearing: $F_{1,15}$=6.846; p=0.01<br>Sociability index: $F_{1,15}$=6.678; p=0.01	6-months<br>WT=9, MPS-IIIA=8
**2c**	χ2 test=0.04	
**2e**	Repeated measures ANOVA (2 levels: training and test; Factor: genotype, 2 levels: WT and MPS-IIIA)<br>Genotype $F_{1,15}$=2.37; p=0.13; % freezing $F_{1,15}$=83.1; p<0.0001; Genotype x % freezing $F_{1,15}$=0.93; p=0.34	
**3a'-a'''; 3b'-b'''**	One-way ANOVA (Factor: genotype, 2 levels: WT, MPS-IIIA)<br>LAMP-1 2 months $F_{1,12}$=8.952; p=0.01<br>LC3-II 8 months $F_{1,7}$=11.888; p=0.02<br>p62 8 months $F_{1,7}$=8.74; p=0.02<br>LAMP-1 8 months $F_{1,9}$=74.33; p<0.0001	2-months<br>WT=7, MPS-IIIA=7;<br>8-months<br>WT=3-5, MPS-IIIA=5-6
**3c**	One-way ANOVA (Factor: genotype, 2 levels: WT, MPS-IIIA)<br>$F_{1,12}$=0.066; p=0.8	WT=7, MPS-IIIA=6
**3d-f**	Repeated measures ANOVA (3 levels: nACC, DMS and DLS; Factor: genotype; 2 levels: WT and MPS-IIIA)<br>D2R: Genotype $F_{1,12}$=4.67; p=0.07; Subregion $F_{2,12}$=0.36; p=0.69; Genotype x Subregion $F_{2,12}$=0.36; p=0.69<br>SYN: Genotype $F_{1,12}$=3.7; p=0.10; Subregion $F_{2,12}$=0.005; p=0.99; Genotype x Subregion $F_{2,12}$=0.005; p=0.99<br>PSD-95: Genotype $F_{1,12}$=4.55; p=0.07; Subregion $F_{2,12}$=0.28; p=0.75; Genotype x Subregion $F_{2,12}$=0.28; p=0.75	WT=4, MPS-IIIA=4
**3g**	Two-way ANOVA (Factor: genotype, 2 levels: WT, MPS-IIIA; Factor: treatment; 2 levels: vehicle, α-MPT)<br>Genotype $F_{1,18}$=0.758; p=0.39; Treatment $F_{1,18}$=88.150; p<0.0001; Genotype x treatment $F_{1,18}$=11.990; p=0.002	WT veh= 6; MPS-IIIA veh=7; WT α-MPT 5h=4; MPS-IIIA α-MPT 5h= 5
**4b-d**	Repeated measures ANOVA (2 levels: SN and VTA; Factor: genotype, 2 levels: WT and MPS-IIIA)<br>PV+ neurons: Genotype $F_{1,6}$=10.838; p=0.01; Subregion $F_{1,6}$=0.018; p=0.89; Genotype x Subregion $F_{1,6}$=0.018; p=0.89<br>NeuN+ neurons: Genotype $F_{1,6}$=9.58; p=0.02; Subregion $F_{1,6}$=0.058; p=0.81; Genotype x Subregion $F_{1,6}$=0.058; p=0.81	WT=4, MPS-IIIA=4
**5a-c**	Repeated measures ANOVA (2 levels: SN and VTA; Factor: genotype, 2 levels: WT and MPS-IIIA)<br>One-way ANOVA (Factor: genotype, 2 levels: WT, MPS-IIIA)<br>TH striatum $F_{1,5}$=7.890; p=0.03	8-months<br>WT=3-4, MPS-IIIA=4
**5d-e**	One-way ANOVA (Factor: genotype, 2 levels: WT, MPS-IIIA)<br>2 months: $F_{1,6}$=0.51; p=0.5<br>8 months: $F_{1,8}$=170.757; p<0.0001	2-months<br>WT=4, MPS-IIIA=4<br>8-months<br>WT=5, MPS-IIIA=5
**6c-c''**	Two-way ANOVA (Factor: genotype, 2 levels: WT, MPS-IIIA; Factor: DIV; 2 levels: DIV4, DIV14)<br>p62: Genotype $F_{1,12}$=11.61; p=0.0052; DIV $F_{1,12}$=29.76; p=0.0001; Genotype x DIV $F_{1,12}$=13.8; p=0.0029	WT=3-4, MPS-IIIA=3-4

	LAMP-1: Genotype $F_{1,12}$=22.9; p=0.0004; DIV $F_{1,12}$=11.73; p=0.005; Genotype x DIV $F_{1,12}$=3.6; p=0.089<br>TUNEL: Genotype $F_{1,8}$=2.91; p=0.12; DIV $F_{1,8}$=39.52; p=0.0002; Genotype x DIV $F_{1,8}$=7.2; p=0.027	
**7b-c**	Two-way ANOVA (Factor: genotype, 2 levels: WT, MPS-IIIA; Factor: DIV; 3 levels: DIV4, DIV7, DIV14)<br>TH: Genotype $F_{1,48}$=0.336; p=0.56; DIV $F_{2,48}$=2.59; p=0.08; Genotype x DIV $F_{2,48}$=17.1; p<0.0001<br>Tuj1: Genotype $F_{1,44}$=6.150; p=0.01; DIV $F_{2,44}$=3.50; p=0.038; Genotype x DIV $F_{2,44}$=15.8; p<0.0001	WT=7-9; MPS-IIIA=7-8
**8a'-c'**	Two-way ANOVA (Factor: genotype, 2 levels: WT, MPS-IIIA; Factor: DIV; 2 levels: DIV4, DIV14)<br>p62: Genotype $F_{1,14}$=5.4; p=0.03; DIV $F_{1,14}$=19.05; p=0.0008; Genotype x DIV $F_{1,14}$=5.2; p=0.03<br>LAMP-1: Genotype $F_{1,8}$=38.76; p=0.0003; DIV $F_{1,8}$=10.03; p=0.01; Genotype x DIV $F_{1,8}$=9.2; p=0.01<br>TUNEL: Genotype $F_{1,7}$=119.2; p<0.0001; DIV $F_{1,7}$=262.32; p<0.0001; Genotype x DIV $F_{1,7}$=120.2; p<0.0001	WT=3-4, MPS-IIIA=3-5
**9b**	One-way ANOVA (Factor: genotype; 2 levels: WT, MPS-IIIA)<br>$F_{1,4}$=9.642; p=0.0360	WT=3, MPS-IIIA=3
**10c'**	Two-way ANOVA (Factor: genotype, 2 levels: WT, MPS-IIIA; Factor: Treatment; 3 levels: veh, HS 25, HS40) Genotype $F_{1,24}$=3.59; p=0.06; Treatment $F_{2,24}$=0.44; p=0.64; Genotype x treatment $F_{2,24}$=0.073; p=0.93	WT n=5; WT HS25 n=5; WT HS40 n=4; MPS-IIIA n=6; MPS-IIIA HS25 n=5; MPS-IIIA HS40 n=5
**10d**	Two-way ANOVA (Factor: genotype, 2 levels: WT, MPS-IIIA; Factor: Treatment; 2 levels: veh, HS 50) Genotype $F_{1,11}$=12.325; p=0.0056; Treatment $F_{1,11}$=10.534; p=0.0088; Genotype x treatment $F_{1,11}$=9.597; p=0.013	WT n=4; WT HS50 n=4; MPS-IIIA n=3; MPS-IIIA HS50 n=4
**11a**	One-way ANOVA (Factor: Treatment, 3 levels: untreated, HS-WT, HS-MPS) $F_{2,6}$=67.75; p<0.0001	Untreated n=3; WT n=3; MPS-IIIA n=3
**12a-a''**	One-way ANOVA (Factor: genotype, 2 levels: WT, MPS-II)<br>LC3-II: $F_{1,7}$=0.044; p=0.84<br>p62: $F_{1,7}$=0.95; p=0.34<br>LAMP1: $F_{1,7}$=85.85; p<0.0001	WT= 4; MPS-II=5

Supplementary Table 3. Detailed information for the supplementary figures.

SUPPLEMENTARY METHOD

Experimental design of behavioural characterization of MPS-IIIA and MPS-II. We tested MPS-IIIA, MPS-II and their WT littermate mice at different time points (MPS-IIIA: at 2-, 6- and 8-months-old and MPS-II: at 1-month-old) in a battery of behavioural tasks to assess autistic-like and dementia-like behaviours (related to Fig. 1, Fig. 8, Supplementary Fig. 2). At the end of the behavioural tasks, mice were used for biochemical (TH, dopamine receptors, DA content etc.) and histological characterization (related to Fig. 2a-e', Fig. 4a-a', Fig. 8f-g', Supplementary Fig. 3a-f, Supplementary Fig. 4, Supplementary Fig. 5, Supplementary Fig. 12). An experimental design scheme was reported in the Supplementary Figure 13.

Experimental design of SCH-23390/Haloperidol treatment

WT and MPS-IIIA animals were used to study the effects of SCH-23390 and haloperidol on autistic-like behavioural symptoms (related to Fig. 3b-b'' and Fig. 3c-c''). The first and the second cohort of mice were treated with SCH-23390 0.03 and 0.06 mg/kg respectively. A single animal received vehicle or SCH-23390 and was tested in the social dominance tube test and immediately after in the social novelty test. The following day each mouse was re-injected with the same treatment and tested in the open field test to measure both locomotor and stereotyped behaviour. Ten/fifteen days apart, mice were re-injected according to a Latin square design[1]. A third cohort of mice was used to test the effects of different doses of haloperidol (0.05 and 0.1 mg/kg), following the same experimental design, but were injected three times. An experimental scheme was reported in the Supplementary Figure 14.

Odour task

A small group of WT and MPS-IIIA mice at 2-months of age was used to test odour discrimination, using the same apparatus and the same habituation/dishabituation protocol used for the social interaction test (related to Supplementary Fig. 1). The odours were presented with cotton-tipped wooden applicators and were lemon or pastiera (an Italian cake). For the five-minute habituation phase, we put two identical odours at each end of the arm. During the five-minute test phase, the odours were replaced with a familiar or a new one. We measured the time spent to explore the odours, using the videotracking system ANY-MAZE (Stoelting, USA) and then analysed the preference of the new odour compared to the familiar one.

Induced dopaminergic neurons

Briefly, MEFs were isolated from embryonic day E14.5 from WT and MPS-IIIA embryos using trypsin 0.25%. Then, cells were infected with a mixture of doxycycline (dox)- inducible lentiviruses expressing for *Nurr1*, *Mash1* and *Lmx1a* as previously described[2,3]. For pyramidal neurons, a mixture of doxycycline (dox)- inducible lentiviruses expressing for *Ngn2*, *Myth1*, *Brn2* and *Mash1*[4] was used. 16–20 h after infection cells were switched into fresh MEFs media containing doxycycline (2mg/ml). After 48 h, media was replaced with neuronal inducing media (DMEM/F12, 25 mg/ml insulin, 50 mg/ml transferrin, 30 nM sodium selenite, 20nM progesterone, 100nM putrescine and penicillin/streptomycin containing doxycycline). At DIV4, 7 and 14 the cells were fixed in PFA 4% and stained for TH, Tuj1, MAP2, p62, LAMP-1 or TUNEL. Images were taken using a confocal microscope (Zeiss LSM 800) and counted using ImageJ software.

FGF2/HS-dependent mitogenic activity (BaF32 assay)

BaF32 cells, which require an addition of exogenous HS to grow[5], were cultured in RPMI 1640 (Invitrogen) supplemented with 10% fetal bovine serum (FBS) and 1 ng/mL interleukin-3 at 37°C, 5% CO2. 50 000 cells/well were cultured in RPMI medium supplemented with 10% horse serum. Under these conditions, FGF2 (50 ng/mL) and HS (100 µg/mL) were added. After 46 h at 37˚C, cells were used to measure the proliferation rate, evaluated with the MTS assay (see Materials and Methods).

Dopamine detection*: *calibration curve.

The application of an appropriate bias on the gate can modulate the current of the channel altering the conductive state of PEDOT:PSS through injection of ions into the bulk of the film. The device was calibrated with DA solutions at known concentration to obtain a calibration curve. Specifically, we evaluated the response of the device for the following dopamine concentrations (μM): 1, 2, 3, 4, 5, 10, 20. The calibration curve was reported in the Supplementary Figure 15.

SUPPLEMENTARY FIGURES

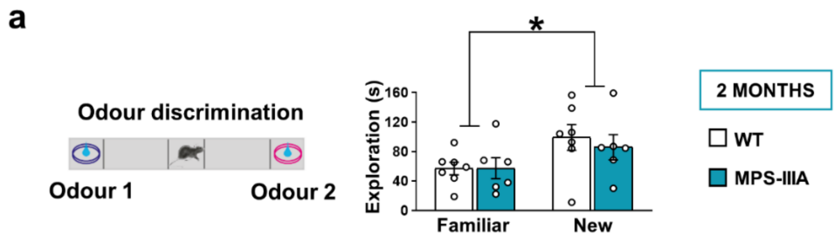

Supplementary Figure 1. (a) In the odour discrimination test, no difference was observed between WT and MPS-IIIA 2-month-old mice. Histograms represent mean ± S.E.M. *p<0.05, new *vs* familiar, within group.

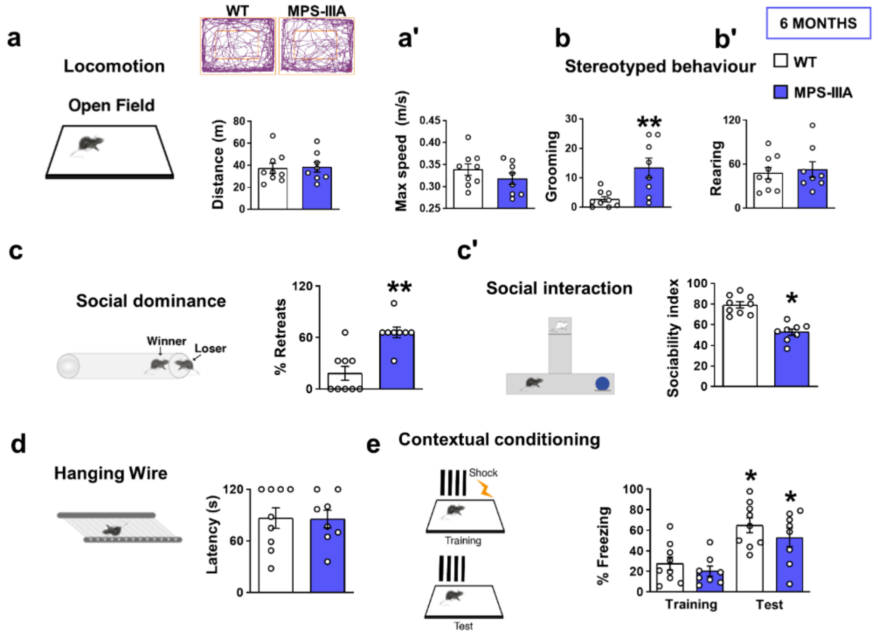

Supplementary Figure 2. (a-a') No differences were present in the locomotion in 6-month-old MPS-IIIA mice, compared to WT littermates. Walking pattern track plots are shown. Histograms represent mean ± S.E.M. **(b-b')** 6-month-old MPS-IIIA mice manifested increased grooming, but did not manifest increased rearing. Histograms represent mean ± S.E.M. ** p<0.05 *vs* WT, between groups. **(c-c')** 6-month-old MPS-IIIA showed an increased percentage of retreats in the social tube test and a decreased sociability index. Histograms represent mean ± S.E.M. **p<0.05 *vs* WT, between groups. **(d-e**) No significant differences were found in the hanging wire and fear conditioning tests between 6-month-old WT and MPS-IIIA mice. Histograms represent mean ± S.E.M. *p<0.05, test *vs* training, within group.

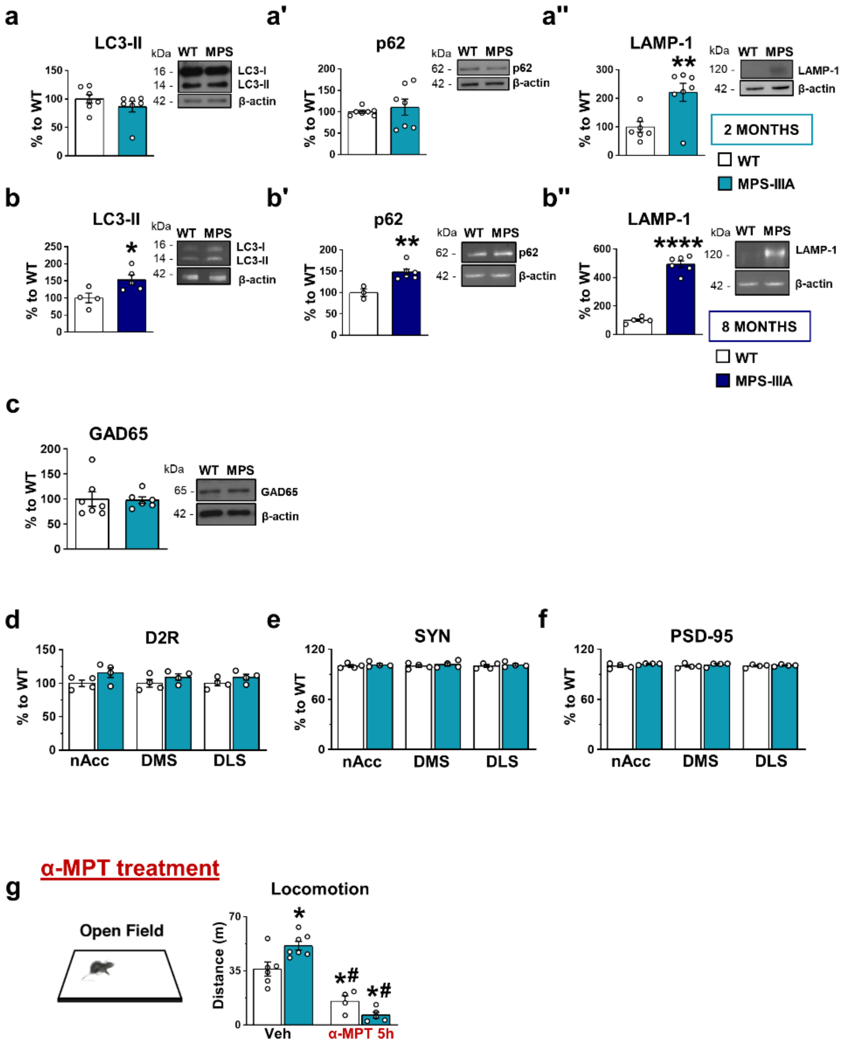

Supplementary Figure 3. (a-a'') Levels of LC3-II, p62 and LAMP-1 in the striatum of 2-month-old WT and MPS-IIIA mice. A Representative WB for each condition is presented. Histograms represent mean ± S.E.M. **p<0.01 *vs* WT, between groups. **(b-b'')** Levels of LC3-II, p62 and LAMP-1 in the striatum of 8-month-old WT and MPS-IIIA mice. A representative WB for each condition is presented. Histograms represent mean ± S.E.M. * p<0.05, **p<0.01, ****p<0.0001 *vs* WT, between groups. **(c)** No significant difference was found in the levels of GAD65 in the striatum of 2-month-old WT and MPS-IIIA mice. A representative WB for each condition is presented. Histograms represent mean ± S.E.M. **(d-f)** Quantification (relative to immunofluorescence showed in Fig. 2e') of total D2R, SYN and PSD-95 showed no change in the expression of these proteins between WT and MPS-IIIA. Histograms represent mean ± S.E.M. **(g)** α-MPT produced dramatic sedative effects in both WT and MPS-IIIA mice 5 hours after injection. Histograms represent mean ± S.E.M. *p<0.05 *vs* WT, between groups; # p<0.05 vs vehicle, within groups.

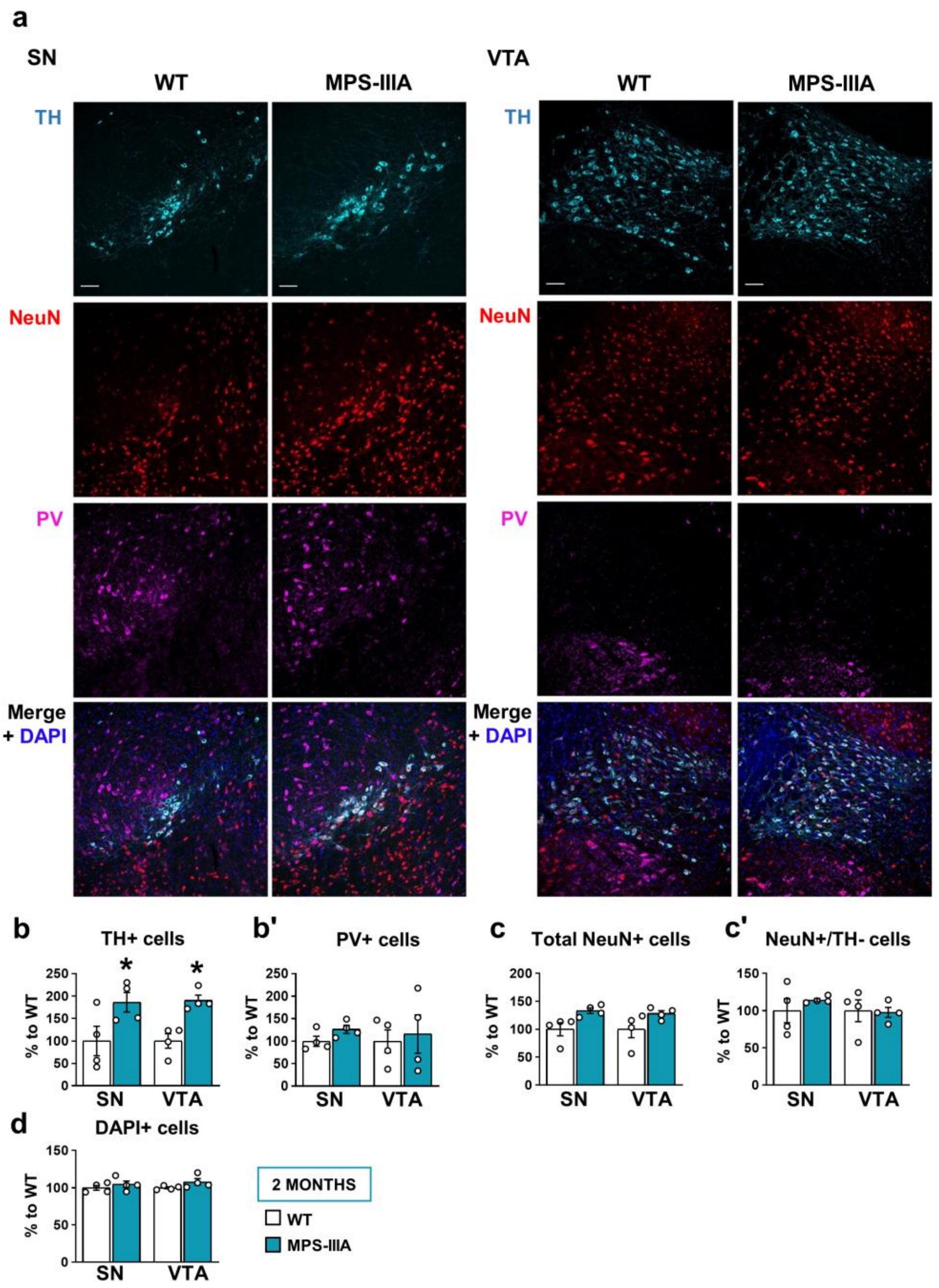

Supplementary Figure 4. (a-d) Immunofluorescence analysis confirmed the increased number of TH+ cells in SN and VTA of MPS-IIIA mice, compared to WT. There was no significant difference in the number of PV+ neurons or in the number of DAPI nuclei between WT and MPS-IIIA. The number of total NeuN+ neurons was also increased. However, when considering only the NeuN+ neurons that were not colocalized with TH, no significant differences were observed between WT and MPS-IIIA. Scale bar: 100 µm. A representative staining for each condition is presented. Histograms represent mean ± S.E.M. *p<0.05 *vs* WT, between group.

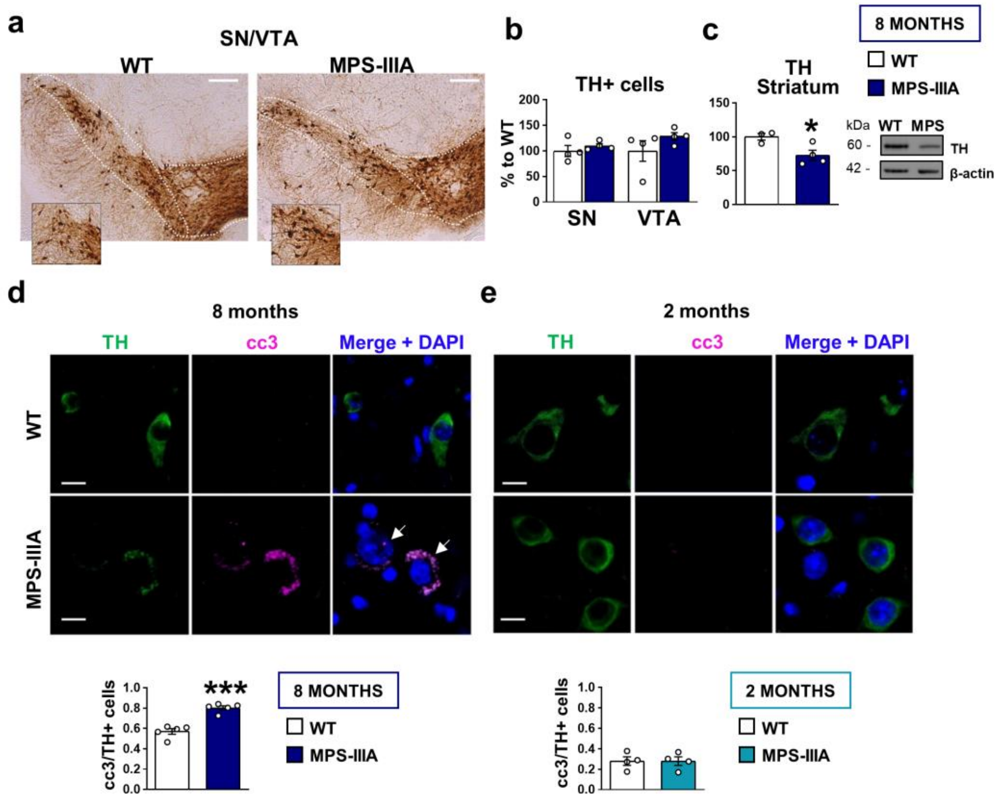

Supplementary Figure 5. (a-c) No significant differences were observed in the number of TH+ neurons in 8-month-old MPS-IIIA mice compared to WT. A representative staining for each condition is presented. Scale bar: 100 µm. TH levels in the striatum of MPS-IIIA mice at 8 months of age is reduced. A representative WB for each condition is presented. Histograms represent mean ± S.E.M. *p<0.05 *vs* WT, between groups. **(d-e)** 8-month-old MPS-IIIA showed an increased number of cleaved caspase 3 (cc3+) neurons suggesting the presence of apoptotic events in MPS-IIIA mice. No signs of cc3 were detected in 2-month-old MPS-IIIA mice. A representative staining for each condition is presented. Scale bar: 10 µm. Histograms represent mean ± S.E.M. ***p<0.001*vs* WT, between groups.

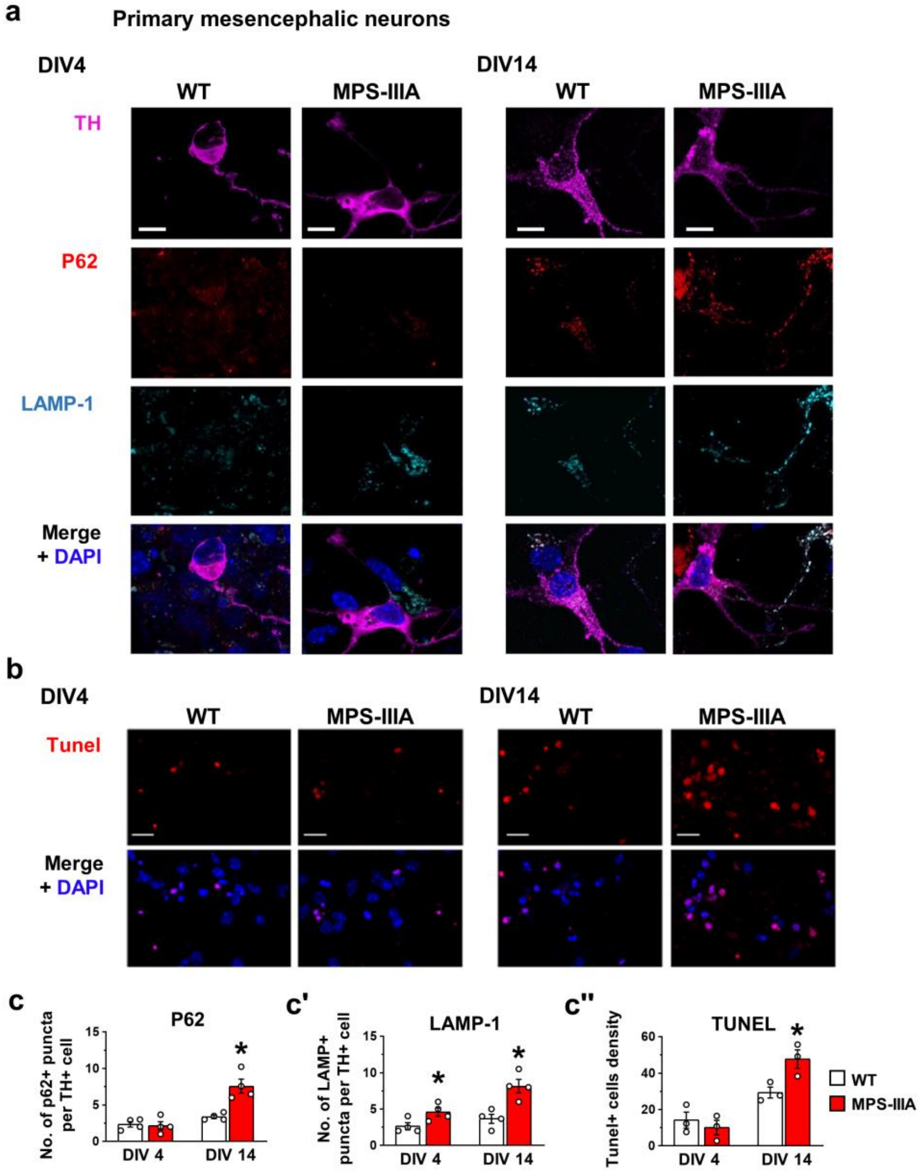

Supplementary Figure 6. (a-c'') Immunofluorescence analysis revealed that there was an increase in the number of p62+ and LAMP-1+ spots in MPS-IIIA primary neurons at DIV14 but not at DIV4, associated with an increased density of TUNEL+ neurons. A representative staining for each condition is presented. Scale bar: 10 µm Histograms represent mean ± S.E.M. *p<0.05 *vs* WT, between groups.

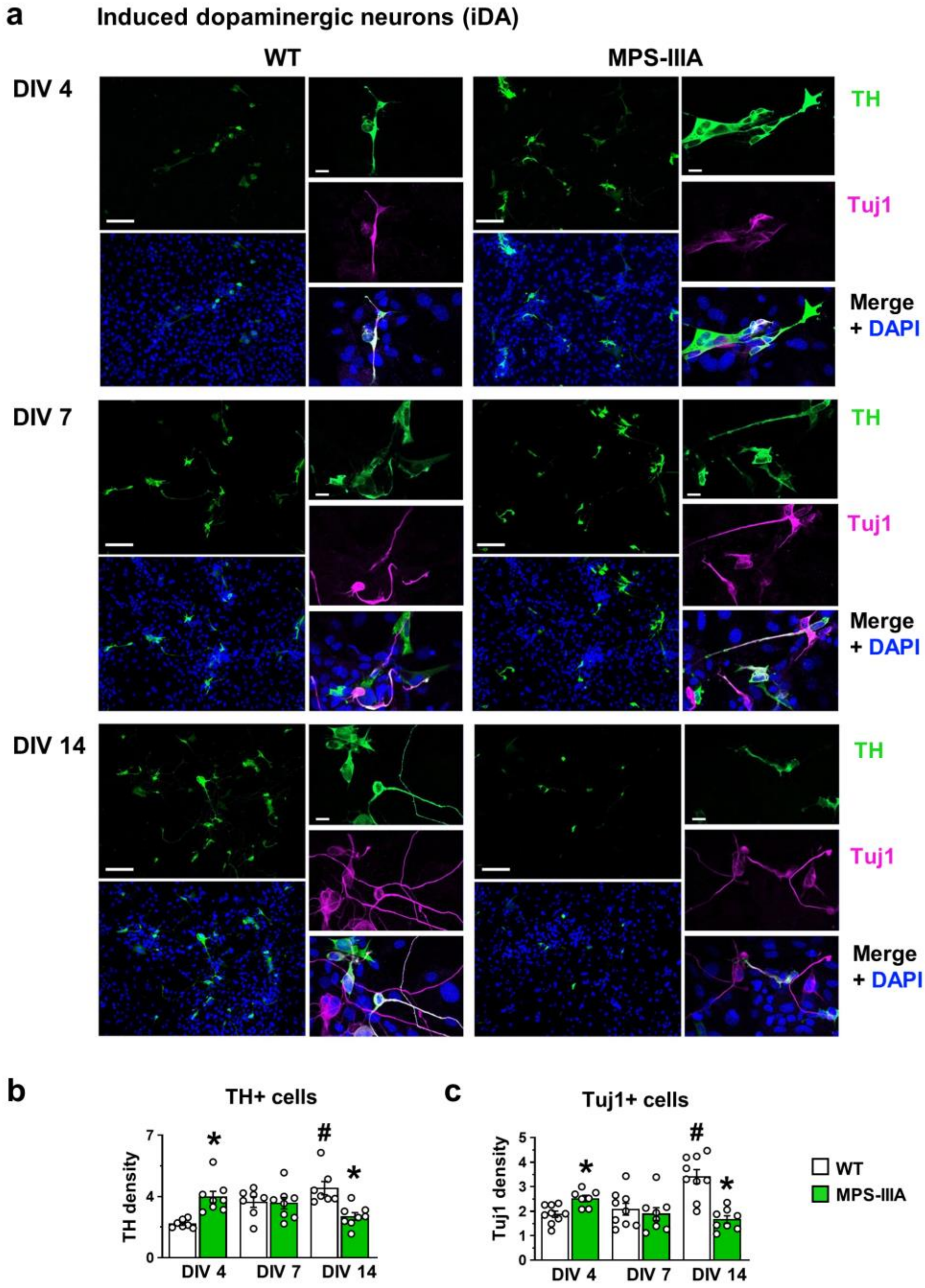

Supplementary Figure 7. (a-b) Induced dopaminergic neurons (iDA) from MPS-IIIA embryonic fibroblasts (MEF) showed a higher percentage of TH+ cells at DIV4 and a progressive loss at DIV14. Scale bar: 75 µm. Histograms represent mean ± S.E.M. *p<0.05 *vs* WT, between groups; # p<0.05 *vs* MPS-IIIA veh, within group. **(c)** Tuj1+ neurons were increased at DIV4, while were reduced at DIV14 in MPS-IIIA cells. Histograms represent mean ± S.E.M. *p<0.05 *vs* WT, between groups; # p<0.05 *vs* MPS-IIIA veh, within group.

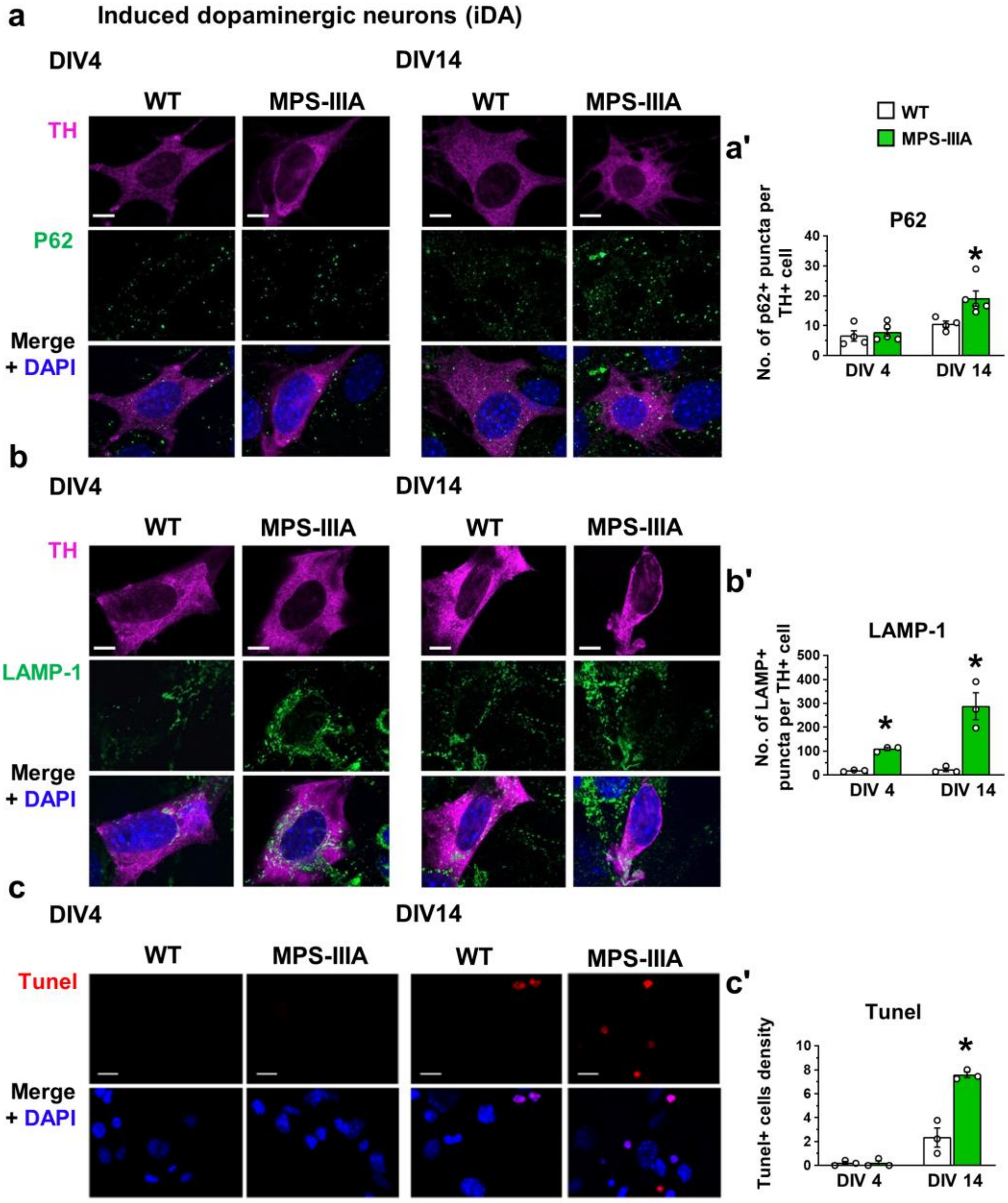

Supplementary Figure 8. (a-c') Immunofluorescence analysis revealed that there was an increase in the number of p62+ and LAMP-1+ spots in MPS-IIIA iDA at DIV14, but not at DIV4. This impairment was associated with an increased density of TUNEL+ neurons. A representative staining for each condition is presented. Scale bar: 10 µm Histograms represent mean ± S.E.M. *p<0.05 *vs* WT, between groups.

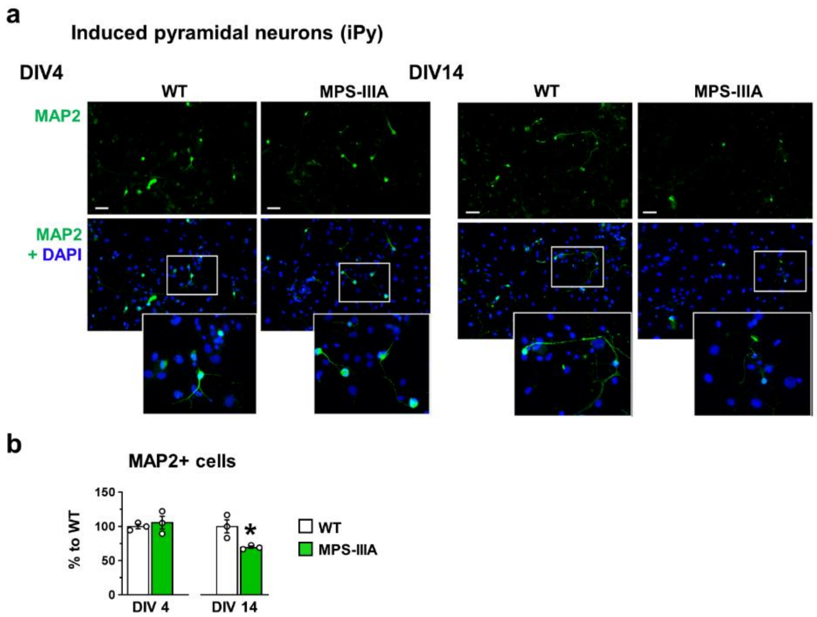

Supplementary Figure 9. (a-b) Induced pyramidal neurons from WT and MPS-IIIA MEFs. At DIV4, there were no difference in the number of MAP2+ cells. At DIV14 a significant decrease was detected in MPS-IIIA cells. A representative staining for each condition is presented. Scale bar: 75 µm. Histograms represent mean ± S.E.M. *p<0.05 *vs* WT, between groups.

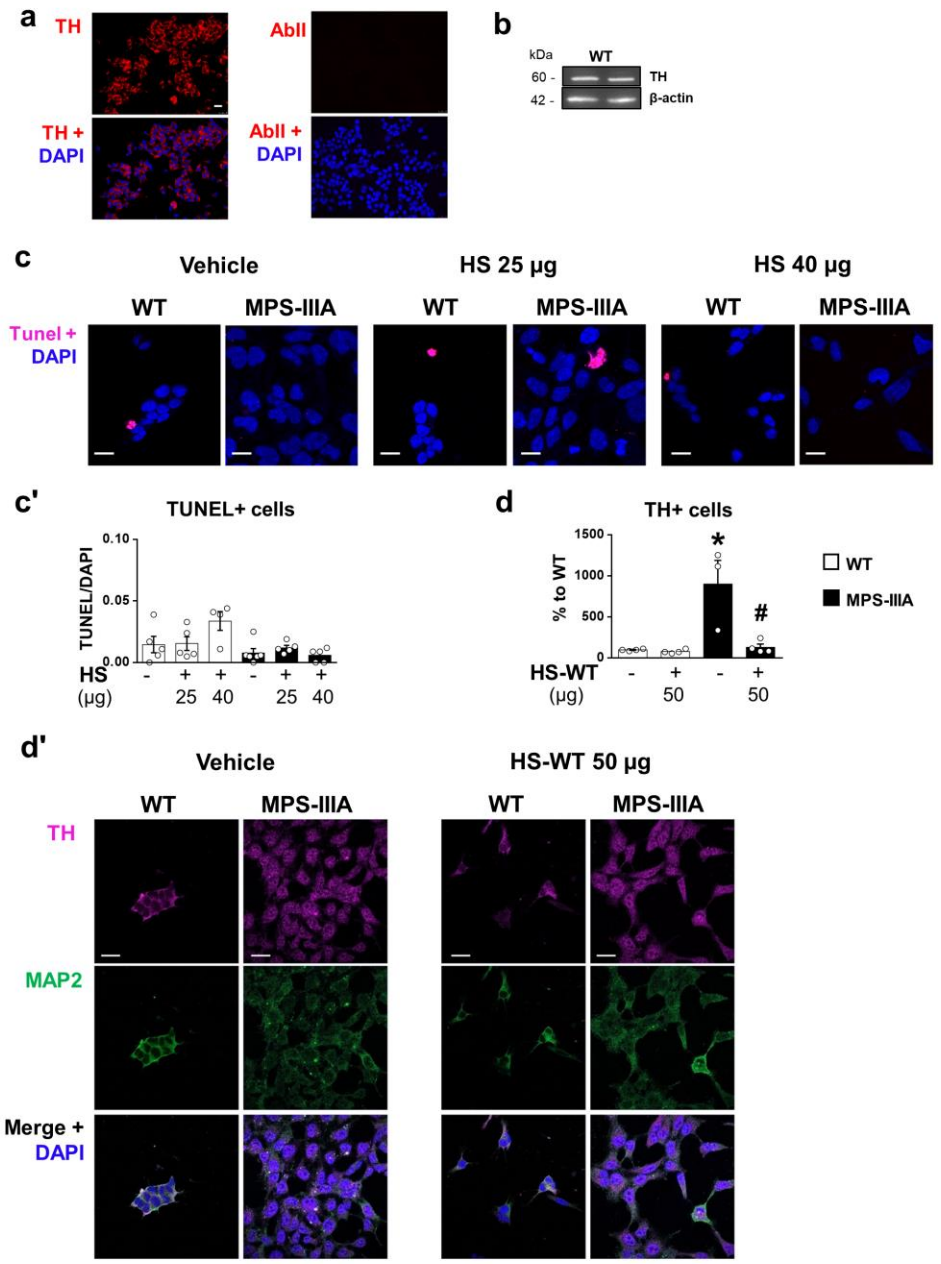

Supplementary Figure 10. (a-b) Representative immunofluorescence and WB verifying the expression of TH in the SH-SY5Y cell line. Panel A also shows staining with the secondary antibody alone (Ab II, negative control). A representative staining for each condition is presented. Scale bar: 50 µm.

(c-c') TUNEL staining after HS treatment in WT and MPS-IIIA SH-SY5Y cells showed no difference between treatments. A representative staining for each condition is presented. Scale bar: 10 µm.

(d-d') WT-HS reduces TH+ cells in MPS-IIIA SH-SY5Y. Histograms represent mean ± S.E.M. A representative staining for each condition is presented. Scale bar: 10 µm. *p<0.05 *vs* WT, between groups; # p<0.05 vs vehicle, within groups.

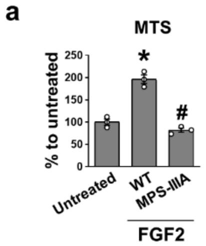

Supplementary Figure 11. (a) MTS assay after treatment with HS extracts from WT or MPS-IIIA (100 µg/mL) brains revealed that MPS-IIIA extracts failed to induce proliferation mediated by FGF2 (50 ng/mL) in BaF32 cells, differently from WT. Histograms represent mean ± S.E.M. *p<0.05 *vs* untreated, between groups; #p<0.05 *vs* WT, within group.

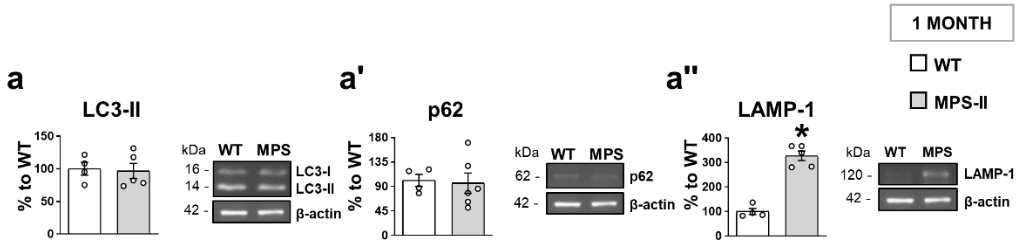

Supplementary Figure 12. (a-a'') Levels of LC3-II, p62 and LAMP-1 in the striatum of 1-month-old WT and MPS-II mice. Representative WB for each condition is presented. Histograms represent mean ± S.E.M. *$p<0.05$ *vs* WT, between groups.

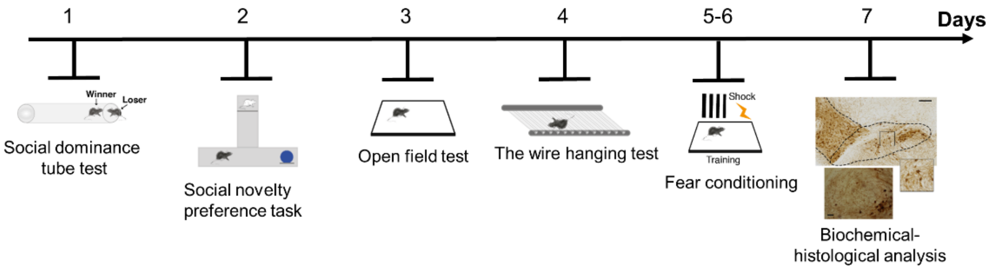

Supplementary Figure 13. Experimental design of behavioural characterization of MPS-IIIA and MPS-II.

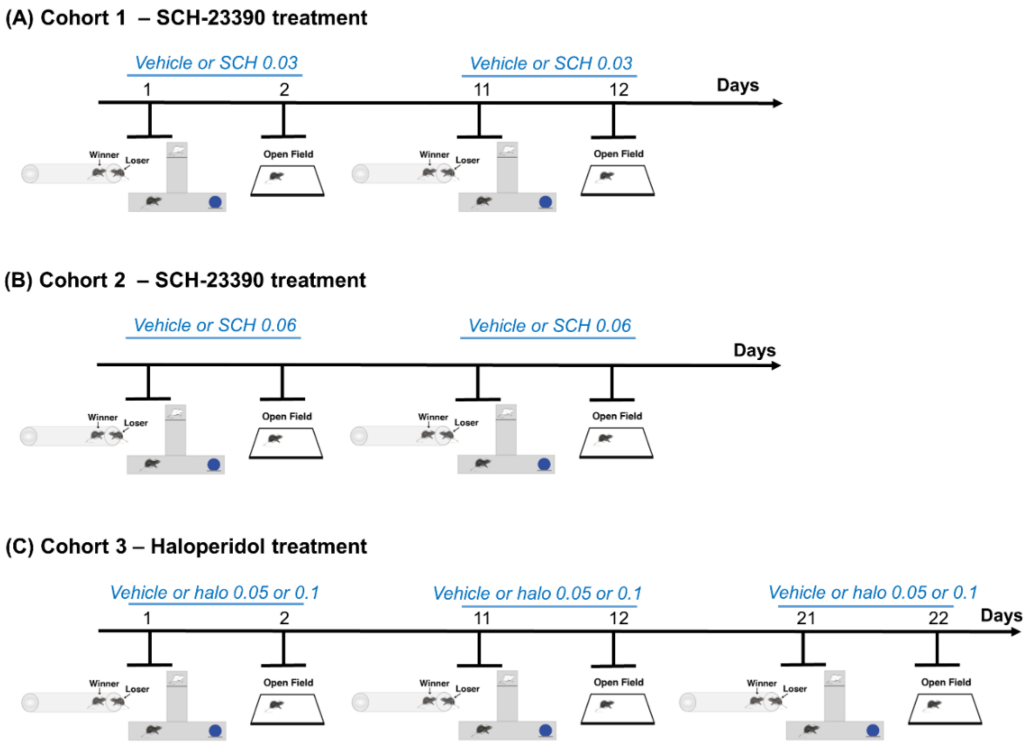

Supplementary Figure 14. Experimental design of SCH-23390/Haloperidol treatment.

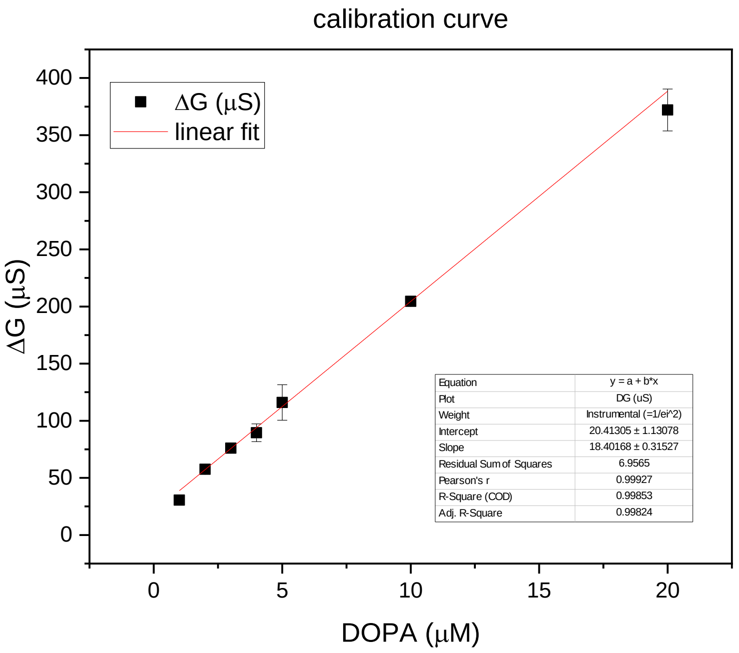

Supplementary Figure 15. Calibration curve of dopamine detection.

SUPPLEMENTARY REFERENCES

1. Robledo, P. Effects of lesions of the nucleus basalis magnocellularis on the acquisition of cocaine self-administration in rats. *Eur. J. Neurosci.* (1998) doi:10.1046/j.1460-9568.1998.00204.x.
2. De Gregorio, R. *et al.* miR-34b/c Regulates Wnt1 and Enhances Mesencephalic Dopaminergic Neuron Differentiation. *Stem Cell Reports* (2018) doi:10.1016/j.stemcr.2018.02.006.
3. Caiazzo, M. *et al.* Direct generation of functional dopaminergic neurons from mouse and human fibroblasts. *Nature* (2011) doi:10.1038/nature10284.
4. Miskinyte, G. *et al.* Direct conversion of human fibroblasts to functional excitatory cortical neurons integrating into human neural networks. *Stem Cell Res. Ther.* (2017) doi:10.1186/s13287-017-0658-3.
5. Huynh, M. B. *et al.* Glycosaminoglycans from Alzheimer's disease hippocampus have altered capacities to bind and regulate growth factors activities and to bind tau. *PLoS One* (2019) doi:10.1371/journal.pone.0209573.
