# Supplementary material for: Altered heparan sulfate metabolism during development triggers dopamine-dependent autistic-behaviours in models of lysosomal storage disorders
Source: Nat Commun. 2021 Jun 9;12:3495. doi: 10.1038/s41467-021-23903-5 (PMC8190083; doi:10.1038/s41467-021-23903-5)
Supplement: Supplementary file 3 — Reporting summary. [file 41467_2021_23903_MOESM3_ESM.pdf]

nature research

Corresponding author(s): Elvira De Leonibus

Last updated by author(s): May 7, 2021

Reporting Summary

Nature Research wishes to improve the reproducibility of the work that we publish. This form provides structure for consistency and transparency in reporting. For further information on Nature Research policies, see our Editorial Policies and the Editorial Policy Checklist.

Statistics

For all statistical analyses, confirm that the following items are present in the figure legend, table legend, main text, or Methods section.

n/a	Confirmed	
☐	☒	The exact sample size (n) for each experimental group/condition, given as a discrete number and unit of measurement
☐	☒	A statement on whether measurements were taken from distinct samples or whether the same sample was measured repeatedly
☐	☒	The statistical test(s) used AND whether they are one- or two-sided<br>*Only common tests should be described solely by name; describe more complex techniques in the Methods section.*
☒	☐	A description of all covariates tested
☐	☒	A description of any assumptions or corrections, such as tests of normality and adjustment for multiple comparisons
☒	☐	A full description of the statistical parameters including central tendency (e.g. means) or other basic estimates (e.g. regression coefficient) AND variation (e.g. standard deviation) or associated estimates of uncertainty (e.g. confidence intervals)
☐	☒	For null hypothesis testing, the test statistic (e.g. F, t, r) with confidence intervals, effect sizes, degrees of freedom and P value noted<br>*Give P values as exact values whenever suitable.*
☒	☐	For Bayesian analysis, information on the choice of priors and Markov chain Monte Carlo settings
☒	☐	For hierarchical and complex designs, identification of the appropriate level for tests and full reporting of outcomes
☒	☐	Estimates of effect sizes (e.g. Cohen's d, Pearson's r), indicating how they were calculated

Our web collection on statistics for biologists contains articles on many of the points above.

Software and code

Policy information about availability of computer code

Data collection	ANY-MAZE Version 3, Stoelting, USA; ImageJ 1.50i; NIS-Elements C 4.20 (Nikon, Florence, Italy); Leica Metamorph imaging software
Data analysis	Statview 5.0 (SAS Institute Inc., North Carolina, USA) and Statistica 10.0 (Statsoft, Tulsa, OK, USA)

For manuscripts utilizing custom algorithms or software that are central to the research but not yet described in published literature, software must be made available to editors and reviewers. We strongly encourage code deposition in a community repository (e.g. GitHub). See the Nature Research guidelines for submitting code & software for further information.

Data

Policy information about availability of data

All manuscripts must include a data availability statement. This statement should provide the following information, where applicable:
- Accession codes, unique identifiers, or web links for publicly available datasets
- A list of figures that have associated raw data
- A description of any restrictions on data availability

Data are available from the corresponding author on reasonable request. Simons Foundation Autism Research Initiative (SFARI) database was consulted (https://gene.sfari.org/database/human-gene/). Source data underlying Figs. 2b, 2d-2d', 2f-f', 8f and Supplementary Figs. 3a-a'', 3b-b'', 3c, 5c, 10b, 12a-a''' are provided as a Source Data file.

Field-specific reporting

Please select the one below that is the best fit for your research. If you are not sure, read the appropriate sections before making your selection.

- [x] Life sciences
- [ ] Behavioural & social sciences
- [ ] Ecological, evolutionary & environmental sciences

For a reference copy of the document with all sections, see nature.com/documents/nr-reporting-summary-flat.pdf

Life sciences study design

All studies must disclose on these points even when the disclosure is negative.

Sample size	Sample size was chosen based on previous literature. Indeed, we used n=6-9 for behavioral study and n=3-6 for histological and biochemical studies, that are numbers compliant to the most of literature (De Risi et al., 2020; Giordano et al., 2018).
Data exclusions	Data exclusions was performed based on the results obtained with Z-score test for outliers. Mice excluded were: an 8-month-old WT mouse (related to Fig. 1) and two mice injected with SCH-23390 (related to Fig. 2f-f'). These information are included in the manuscript.
Replication	The reproducibility of the experimental findings was verified by replicating the same results using different cohorts of animals. All the in vitro experiments were performed at least in triplicate.
Randomization	Mice were allocated into experimental groups in random order.
Blinding	The investigators were blinded to experimental condition (genotype or treatment).

Reporting for specific materials, systems and methods

We require information from authors about some types of materials, experimental systems and methods used in many studies. Here, indicate whether each material, system or method listed is relevant to your study. If you are not sure if a list item applies to your research, read the appropriate section before selecting a response.

Materials & experimental systems

n/a	Involved in the study	
[ ]	[x]	Antibodies
[ ]	[x]	Eukaryotic cell lines
[x]	[ ]	Palaeontology and archaeology
[ ]	[x]	Animals and other organisms
[x]	[ ]	Human research participants
[x]	[ ]	Clinical data
[x]	[ ]	Dual use research of concern

Methods

n/a	Involved in the study	
[x]	[ ]	ChIP-seq
[x]	[ ]	Flow cytometry
[x]	[ ]	MRI-based neuroimaging

Antibodies

Antibodies used	The antibodies used in this study were: anti-TH (1:1000, AB152, Millipore), anti-D1R (1:500, sc-14001, Santa Cruz), anti-D2R (1:500, AB5084P, Millipore), anti-DARPP-32 (1:1000, AB10518, Millipore), anti-P-DARPP-32-phosphoThr34 (1:1000, AB9206, Millipore), anti-BrdU (1:200, NB500-169; Novusbio), anti-LAMP-1 (1:500, sc-19992, Santa Cruz), anti-p62 (1:500, H00008878-M01, Tebu-bio), anti-LC3B (1:1000, NB100-2220, Novus-Bio), anti-GAD65 (1:1000, BK3988S, Cell Signaling), anti-β-actin (1:5000, MAB1501, Millipore), anti-LMX1A (1:1000, AB10533, Millipore), anti-cleaved caspase 3 (1:400, 9661, Cell Signaling); anti-MAP2 (1:400, MA5-12826, Invitrogen), anti-Tuj1 (1:400, MAB1637, Millipore); anti-Flag (1:200, F3165, Sigma); synaptophysin (1:500, 101004, Synaptic System); PSD-95 (1:100, 124011, Synaptic System); Alexa-488 or -546 or -350 secondary donkey anti-IgGs (1:500, Invitrogen Life Technology); IgG peroxidase-labelled antibody (1:200, ab6721, Abcam)
Validation	We used antibodies already validated by previous studies (De Risi et al., 2020; Giordano et al., 2018) or by manufacturer for western blot or immunofluorescence. Moreover, control of specificity of immunolabeling were performed by omission of primary antibodies.

Eukaryotic cell lines

Policy information about cell lines

Cell line source(s)	We used: primary cell lines obtained by mouse embryos (neurons and fibroblasts); CRISPR/Cas9 SH-SY5Y cell line provided by Dr. Jlenia Monfregola and Prof. Andrea Ballabio (TIGEM); Baf32 cell line provided by Prof. Dulce Papy-Garcia (UPEC).
Authentication	Cell lines were not authenticated.

Mycoplasma contamination	Cell lines were not tested for mycoplasma contamination, except for Baf32 cell line that resulted negative for mycoplasma.
Commonly misidentified lines (See ICLAC register)	We did not use misidentified lines.

Animals and other organisms

Policy information about studies involving animals; ARRIVE guidelines recommended for reporting animal research

Laboratory animals	MPS-IIIA (Sgsh -/-), MPS-II (Ids y/−) and respective control littermate WT congenic C57BL/6 mice were used. We tested three different groups of MPS-IIIA and WT littermates mice at different time points (2, 6 and 8 months of age) and a group of MPS-II and WT littermates mice (1-month-old). For histological/cellular studies we also used WT and MPS-IIIA mice at embrionic day E13.5, postnatal day 0 and postnatal day 28. Only male mice were used. We use a small batch of CD1 male mice (1, 2, 6 or 8 months of age) as "stranger" in the social novelty preference task. Mice were group-housed in Plexiglas cages (18 × 35 × 12 cm) with free access to food and water and kept at a temperature range between 20 and 23°C and and 55 ± 5 % relative humidity. All procedures were performed during daylight hours (between 9 AM and 6 PM).
Wild animals	We did not use wild animals.
Field-collected samples	No field-collected samples were used in the study.
Ethics oversight	Animal studies were conducted in accordance with the guidelines and policies of the European Communities Council and were approved by the Italian Ministry of Health.

Note that full information on the approval of the study protocol must also be provided in the manuscript.
